# Genomic profile of Chinese patients with endometrial carcinoma

**DOI:** 10.1186/s12885-023-11382-4

**Published:** 2023-09-20

**Authors:** Jin Li, Xiaoqi Li, Chenlian Quan, Xiaoqiu Li, Chong Wan, Xiaohua Wu

**Affiliations:** 1 Department of Gynecologic Oncology, Fudan University Shanghai Cancer Center, Fudan University, 200032 Shanghai, China; 2 Department of Pathology, Fudan University Shanghai Cancer Center, Fudan University, 200032 Shanghai, China; 3 Department of Oncology, Shanghai Medical College, Fudan University, 200032 Shanghai, China; 4 Precision Medicine Center, Yangtze Delta Region Institute of Tsinghua University, Jiaxing, China

**Keywords:** Endometrial carcinoma, Genomic profile, Somatic alterations, Genomic alterations, ARID1A, DNA damage repair

## Abstract

**Backgrounds:**

Endometrial carcinoma (EC) is one of the most commonly diagnosed gynecologic malignancy in China. However, the genetic profile of Chinese EC patients has not been well established yet.

**Methods:**

In current study, 158 Chinese EC patients were subjected to next-generation sequencing assay (74 took testing of EC-related 20-genes panel, and 84 took the expanded panel). Of the 158 patients, 91 patients were performed germline mutation testing using the expanded panel. Moreover, the public datasets from TCGA and MSKCC were utilized to compare the genomic differences between Chinese and Western EC patients. The proteomic and transcriptomic from CPTAC and TCGA were derived and performed unsupervised clustering to identify molecular subtypes.

**Results:**

Among the 158 patients analyzed, a significant majority (85.4%) exihibited at least one somatic alteration, with the most prevalent alterations occurring in *PTEN*, *PIK3CA*, *TP53*, and *ARID1A*. These genomic alterations were mainly enriched in the PI3K, cell cycle, RAS/RAF/MAPK, Epigenetic modifiers/Chromatin remodelers, and DNA damage repair (DDR) signaling pathways. Additionally, we identified ten individuals (11.0%) with pathogenic or likely pathogenic germline alterations in seven genes, with the DDR pathway being predominantly involved. Compared to Western EC patients, Chinese EC patients displayed different prevalence in *AKT1*, *MET*, *PMS2*, *PIK3R1*, and *CTCF*. Notably, 69.6% of Chinese EC patients were identified with actionable alterations. In addition, we discovered novel molecular subtypes in *ARID1A* wild-type patients, characterized by an inferior prognosis, higher *TP53* but fewer *PTEN* and *PIK3CA* alterations. Additionally, this subtype exhibited a significantly higher abundance of macrophages and activated dendritic cells.

**Conclusion:**

Our study has contributed valuable insights into the unique germline and somatic genomic profiles of Chinese EC patients, enhancing our understanding of their biological characteristics and potential therapeutic avenues. Furthermore, we have highlighted the presence of molecular heterogeneity in ARID1A-wild type EC patients, shedding light on the complexity of this subgroup.

**Supplementary Information:**

The online version contains supplementary material available at 10.1186/s12885-023-11382-4.

## Introduction

Endometrial carcinoma (EC) is one of the most common gynecological cancers worldwide, globally causing an estimated 90,000 deaths in 2018 [1]. Accompanied by the increase of obesity and aging population, the incidence of EC is rising [2]. In China, EC is the second most commonly diagnosed gynecological cancer, with approximately 84,520 new cases reported in 2022 [3]. Endometrioid endometrial carcinoma (EEC) is the most common histologic type, accounting for 85% of all EC cases, and following by serous carcinomas, clear cell carcinoma, and uterine carcinosarcomas [4]. The Cancer Genomic Atlas (TCGA) characterized the genetic landscape of western EC in 2013, and classified patients into four molecular subgroups: *POLE*-mutated (ultramutated), microsatellite instability hypermutated, copy number (CN)-low and high [5]. Remarkably, EEC was present in all four subgroups, while serous carcinoma was mainly found in the CN-high subgroup. Notably, patients in the *POLE*-mutated subgroup exihibted better progression-free survival, whereas those in CN-high subgroup (serous-like) have the poorest prognosis. The molecular classification of EC offers significant targets for the diagnosis and treatment of EC and has been endorsed by the National Comprehensive Cancer Network (NCCN) guideline.

Extensive research has been conducted on the genomic features of western patients with EC. Among them, genomic alteration in *PTEN* and *TP53* were found to be more common, with prevalence ranging from 22 to 65% and 28-63%, respectively [5–8]. Moreover, previous studies have demonstrated the genetic heterogeneity of EC, with the prevalence of genetic alterations being correlated with specific molecular and histopathological subtypes [5, 9]. Recent genomic analyses have also identified crucial potential actionable alterations in EC. For example, a study found that 67% of EC patients had at least one likely therapeutically actionable alteration (excluding RAS mutations). The most frequently identified clinically actionable alterations include *PIK3CA* variant, *PTEN* variant, and *ERBB2* amplification. Employing actionable alteration testing to guide treatment decisions and match patients with the most appropriate clinical trials has shown promising potential to improve outcomes for those with advanced disease [10–12]. Notably, around 47% of EC patients who underwent NGS panel tumor profiling and received therapy tailored to their genomic profile achieved clinical benefit [6, 13].These findings highlight the significance of genomic profiling in guiding treatment strategies and enhancing the management of EC patients.

However, the genomic landscape of Chinese patients with EC remains poorly understood. A recent investigation examined the genomic characteristics of 79 Chinese EEC patients [14], and the results have shed a light on the genomic heterogeneity between Chinese and Western EC patients.

In this study, we aimed to investigate the genomic alterations in a cohort of 158 Chinese EC patients using next-generation sequencing (NGS). We described the basic profile of driver gene alterations in these patients and compared them with counterparts in western cohorts to gain a better understand the molecular feature specific to Chinese EC patients. Additionally, the findings of this study may provide valuable insights for clinicians and researchers in tailoring precision medicine for Chinese EC patients.

## Materials and methods

### Biospecimen collection and clinical data

In our cohort, we collected a total of 121 tissues, including 32 fresh-frozen tumors and 89 formalin-fixed, paraffin-embedded (FFPE) tissues, as well as 37 blood samples, from a total of 158 EC patients for genetic testing. Blood samples were collected primarily due to the unavailability of archived tumor tissue, the inability to obtain fresh tumor tissues and metastatic disease. Additionally, out of the enrolled patients, 91 individuals consented to undergo germline testing for understanding the hereditary characteristics of cancer. This study was approved by the clinical ethics committee of Fudan University Shanghai Cancer Center, Fudan University, and all patients provided written informed consent. To ensure the sample quality, all tumor tissues samples were pathologically assessed to have a tumor content beyond 20%. All the collected samples successfully passed the quality control process and contain sufficient DNA content to enable NGS testing with high efficiency.

### Target next-generation sequencing

The DNA extraction and next-generation sequencing procedures were carried out according to previously established protocols [15]. In brief, DNA extraction from tumor tissue or peripheral blood mononuclear cell (PBMC) samples was performed using the DNeasy Blood & Tissue Kit, while plasma samples were utilized for cell-free DNA (cfDNA) extraction using the QIAamp Circulating Nucleic Acid Kit (both from Qiagen, Inc.). The quantification of DNA was performed using the Qubit 3.0 Fluorometer and the StepOnePlus System, manufactured by Life Technologies, Inc. To achieve fragment sizes of approximately 200 base pairs (bp), 100 ng of genomic DNA (gDNA) from the tumor tissue or PBMC was fragmented using the Covaris E210 system. Subsequently, next-generation sequencing (NGS) was carried out on the tumor or germline gDNA using the Accel-NGS 2 S DNA Library Kit (Swift Biosciences, Inc.) for library preparation and the xGen Lockdown Probes kit (IDT, Inc.) for target enrichment. Custom probes for specific genes were synthesized by IDT, Inc. All samples were subjected to genetic testing, targeting a panel of 20 or 499 genes associated with EC, as listed in Supplementary Table 1. The quantification of the prepared library was performed using the Qubit 3.0 Fluorometer from Life Technologies, Inc., and its quality and fragment size distribution were assessed using an Agilent 2100 Bioanalyzer (Agilent Technologies, Inc.). Paired-end sequencing was conducted on an Illumina Novaseq 6000 platform, manufactured by Illumina Inc., employing 150 bp read lengths. The mean coverage achieved for tumor, PBMC, and cfDNA samples exceeded 1000×, 200×, and 4000×, respectively.

### Data processing

The raw sequencing data were aligned to the reference human genome (UCSC HG19) using the Burrows-Wheeler Aligner. Duplicate sequences were removed, and local realignment was performed. The Genome Analysis Toolkit (GATK) v3.7 was utilized to identify and characterize single nucleotide variations (SNVs) as well as insertions and deletions (INDELs). The ANNOVAR software was employed to annotate the identified variants. Copy number variations (CNVs) were determined using CNVkit, accessible at https://github.com/etal/cnvkit. Variants detected in genomic DNA (gDNA) from PBMC, with an allele fraction (AF) exceeding 25%, were categorized as germline variants. Additionally, variants with a frequency of ≥ 1% in the ExAC (http://exac.broadinstitute.org), 1000 Genomes (http://www.1000genomes.org), or ESP6500 databases (https://evs.gs.washington.edu/EVS) were excluded as benign or likely benign variants. Somatic variants specific to the tumor were obtained by eliminating germline alterations, thus retaining only the variants unique to the tumor.

### Actionable alteration annotating

The functional classification of each somatic mutation was performed according to the interpretation and reporting standards and guidelines recommended by the Association for Molecular Pathology, American Society of Clinical Oncology, and College of American Pathologists (ASCO/CAP). Meanwhile, all identified variants were annotated following the level of evidence established by the OncokB database [16].

### Data sources

The genomic and clinical data Western patients diagnosed with uterine corpus endometrial carcinoma (UCEC), namely UCEC_TCGA and PanCancer Atlas (consisting of 517 patients) was obtained from cbioportal (https://www.cbioportal.org). In addition, a group of 95 patients with UCEC, known as the CPTAC cohort, was chosen as a control cohort of and also derived from cbioportal website. Additionally, ctDNA mutation profile from 44 western EC patients were derived from MSKCC cohort through cbioportal website [17]. Genomic testing of UCEC_TCGA and CPTAC samples was performed using whole exome sequencing, while MSKCC_ctDNA samples underwent analysis using the MSK-IMPACT assay, which encompasses 468 genes. The coverage of genes of the current study can be found in supplementary Table 1.

### Identifying the molecular subtypes of ***ARID1A***wildtype EC patients

In our analysis, we employed the similarity network fusion technique, utilizing the R package “CancerSubtypes” with default parameters, to perform unsupervised clustering on the transcriptomic and proteomic data within the CPTAC cohort [18]. The resulting similarity matrix was then utilized as input for unsupervised clustering using the R package “ConsensusClusterPlus” [19]. Lastly, we employed the random forest algorithm to identify genes associated with the different subtypes or clusters.

### TCGA subtype classification

The four subtypes of endometrial cancer, including POLE, MSI, CNV-high, and CNV-low were adapted from the cbioportal website.

### Statistical analysis

SPSS, GraphPad Prism 9 software, and R language statistical package were performed to statistical analyses. Overall survival (OS) curves were constructed using the Kaplan–Meier method, and the log-rank test was performed. The Chi-Square test and Fisher’s exact test were used to analyze the difference in gene prevalence between different groups. Difference was considered significant if the two-tailed p-value was less than 0.05.

## Results

### Patient characteristics

A total of 158 patients were enrolled in this cohort, with a median age at diagnosis of 56 (range 25–80 years), and EEC (84.81%) was the most common histological type. Among the enrolled patients, 74 (46.84%) were subjected to genetic testing using the 20-gene panel, while the remaining 84 patients (53.16%) underwent testing using the expanded panel of 499 genes, as presented in Table 1 (Table 1).

### Genomic landscape of chinese EC patients

**Fig. 1:**
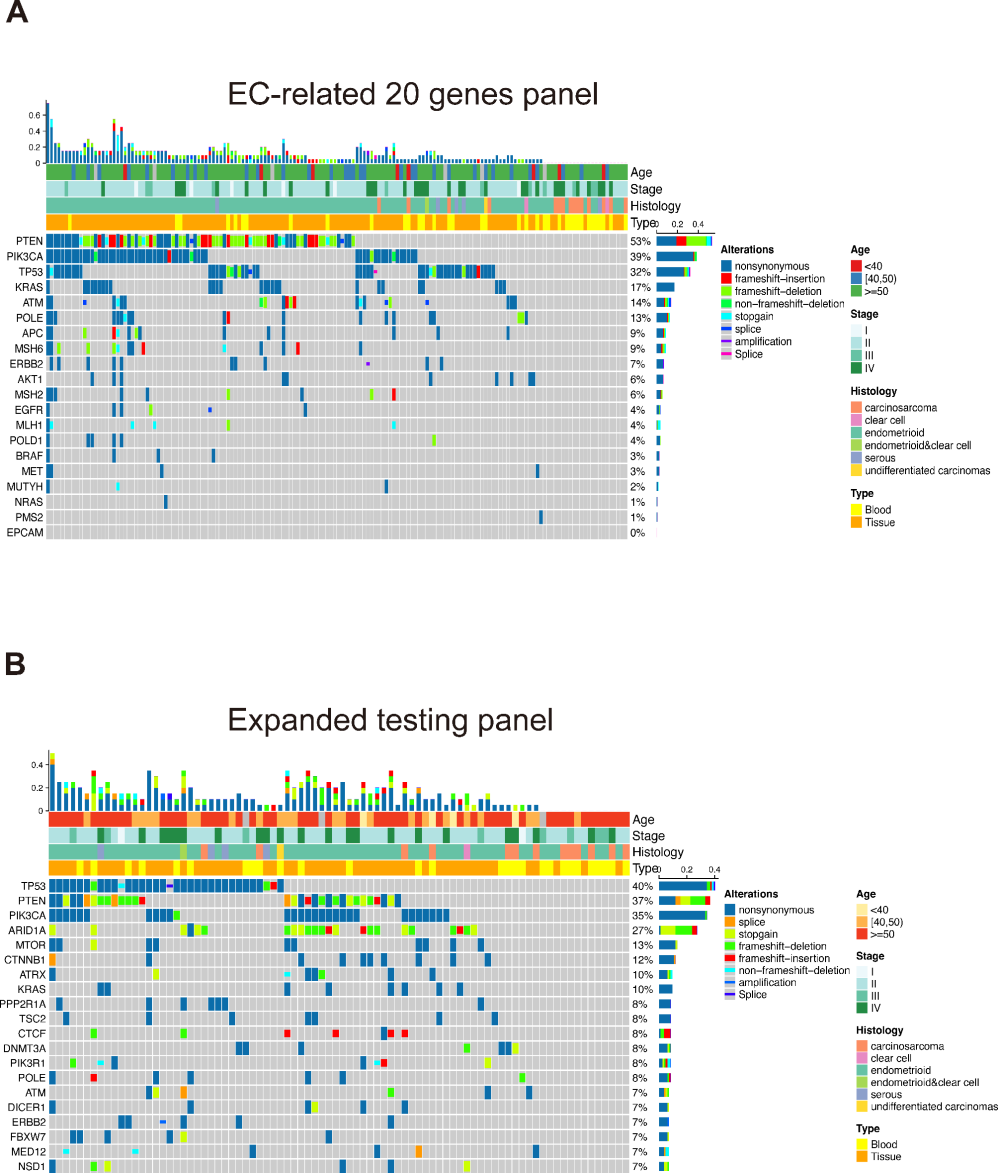
Somatic mutation landscape of Chinese patients with endometrial carcinoma. **(A)** Mutation landscape of 20 genes that all patients have tested (n = 158). **(B)** Mutation landscape of EC patients took the expanded panel testing (n = 84)

All samples underwent deep targeted sequencing of all exons and selected introns of at least 20 selected EC-related genes, and 85.4% (135/158) of them were identified with at least one somatic alteration. The gene with the highest prevalence of alterations was *PTEN* (53.2%, 84/158), following by *PIK3CA* (38.7%, 61/158), *TP53* (32.3%, 51/158), *KRAS* (17.1%, 27/158), *ATM* (13.9%, 22/158), and *POLE* (12.7%, 20/158) (Fig. 1A). in the subset of samples (n = 84) that underwent testing using the expanded panel testing, 82 patients harbored at least one identified genomic alteration. The most prevalent alterations were *TP53* (40.5%, 34/84), *PTEN* (36.9%, 31/84), *PIK3CA* (34.5%, 29/84), *ARID1A* (27.4%, 23/84), *MTOR* (13.1%, 11/84), and *CTNNB1* (11.9%, 10/84) (Fig. 1B). In our cohort, no significantly mutually-exclusive altered genes were identified. However, the common co-altered genes pairs included *PIK3CA* and *PTEN*, *PTEN* and *MSH6*, *PIK3CA* and *POLE*.

The distribution of genomic alterations in *PTEN*, *POLE*, *TP53* and *PIK3CA* were shown in Supplementary Fig. 1. *PTEN* is one of the most frequently mutated genes in our cohort and more than half of EC patients harbored alteration in it. The alteration of *PTEN* distributed relatively throughout the whole protein, most of which are actionable/driver variants (75.7%, 106/140), including R130Q/G/P, K267fs, T319fs. Similarly, *POLE* alterations were dispersed in exon 9–48, including three of V411L and two of P286R. Notably, *TP53* alterations were predominantly enriched in P53 DNA-binding domain. Among *PIK3CA* alterations, a significant proportion were located in exon 2 (23/93), 10 (16/93), and 21 (32/93), including hotspot alterations such as E545G/K and H1047R.

### Signal pathway analysis

**Fig. 2:**
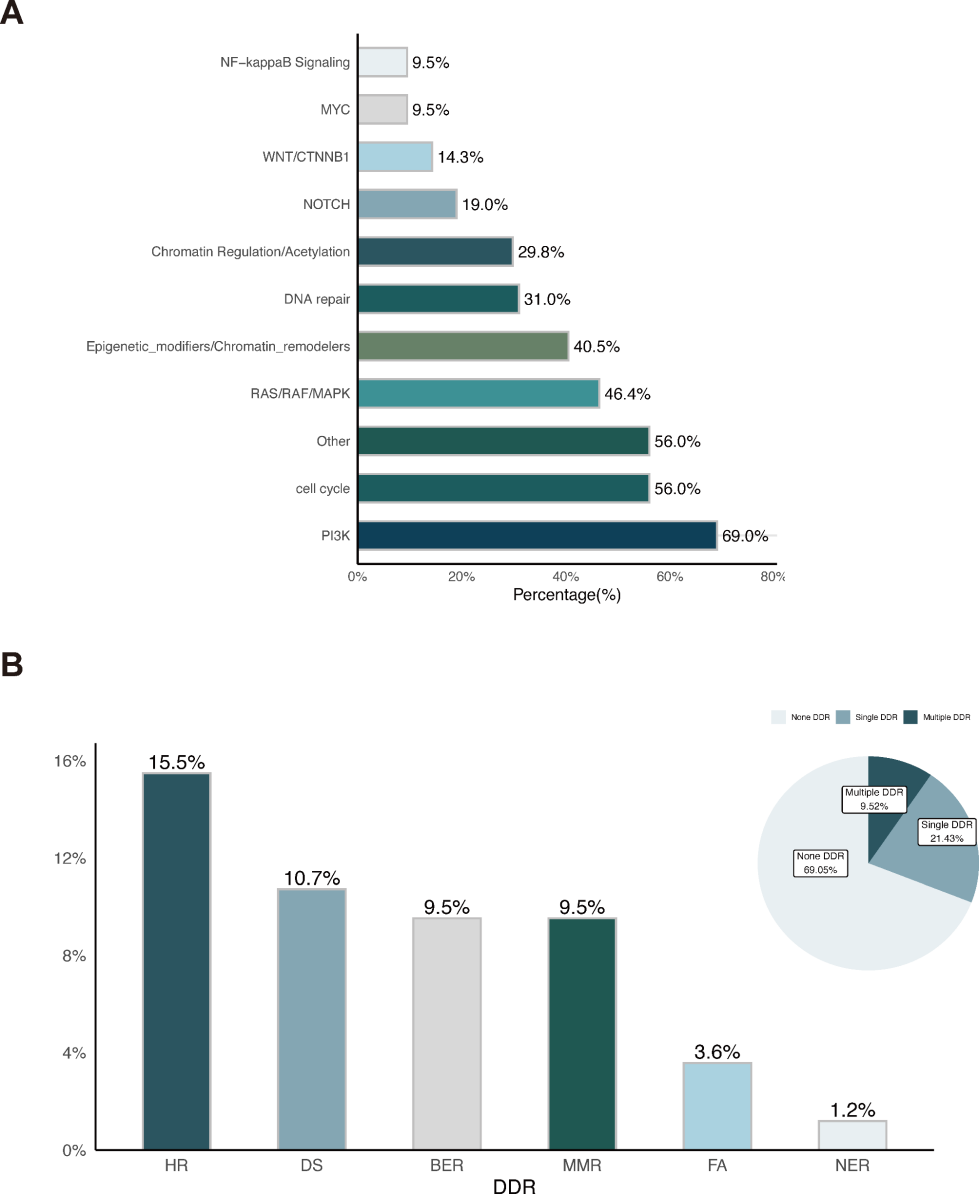
Signaling pathways related to Chinese patients with endometrial carcinoma. **A**, The prevalence of oncogenic and total alterations in specific signal pathways. **B**, The prevalence of altered pathways for DDR. DDR: DNA damage repair; HR: homologous recombination repair, DS: damage sensor, BER: base excision repair, MMR: mismatch repair, FA: Fanconi anemia, NER: nucleotide excision repair

Next, we conducted signal pathway analyses to examine the alterations present in Chinese EC patients who underwent genomic testing using the expanded panel. The analysis revealed that the alterations were most enriched in the following pathways: PI3K (69.0%), cell cycle (56.0%), RAS/RAF/MAPK (46.4%), Epigenetic modifiers/ Chromatin remodelers signaling pathway (40.5%) and DNA damage repair (DDR, 30.1%) (Fig. 2A). Furthermore, 9.52% of Chinese EC patients had multiple DDR alterations, while 21.43% had only one DDR alteration (Fig. 2B). Among the patients with DDR alterations, the most common were homologous recombination repair (HR) and damage sensor (DS), followed by base excision repair (BER), mismatch repair (MMR), Fanconi anemia (FA), and nucleotide excision repair (NER)(Fig. 2B). Moreover, we discovered that patients with DDR pathway alterations exhibited a higher frequency of additional alterations, particularly in genes includin*g MTOR, CTNNB1, ATRX, FAT1*, and *KMT2B* (P < 0.05, Supplementary Fig. 2).

### Germline variants

Among Chinese EC patients in our cohort, a total of ten individuals (11.0%) were identified with pathogenic or likely pathogenic germline mutations in seven genes (Table 2). Notably, these alterations were primarily enriched in in the DDR pathway. Specifically, six patients were identified with pathogenic or likely pathogenic germline in MMR genes (*MLH1*, *MSH2, or MSH6*), with one patient having a co-occurring alteration in *MSH2* and *RAD50*. Additionally, two patients had *BRCA2* mutations, one patient had a *BARD1* mutation, and one patient had a *STK11* mutation.

### Differences in the prevalence of genetic alterations between chinese and western cohorts

**Fig. 3:**
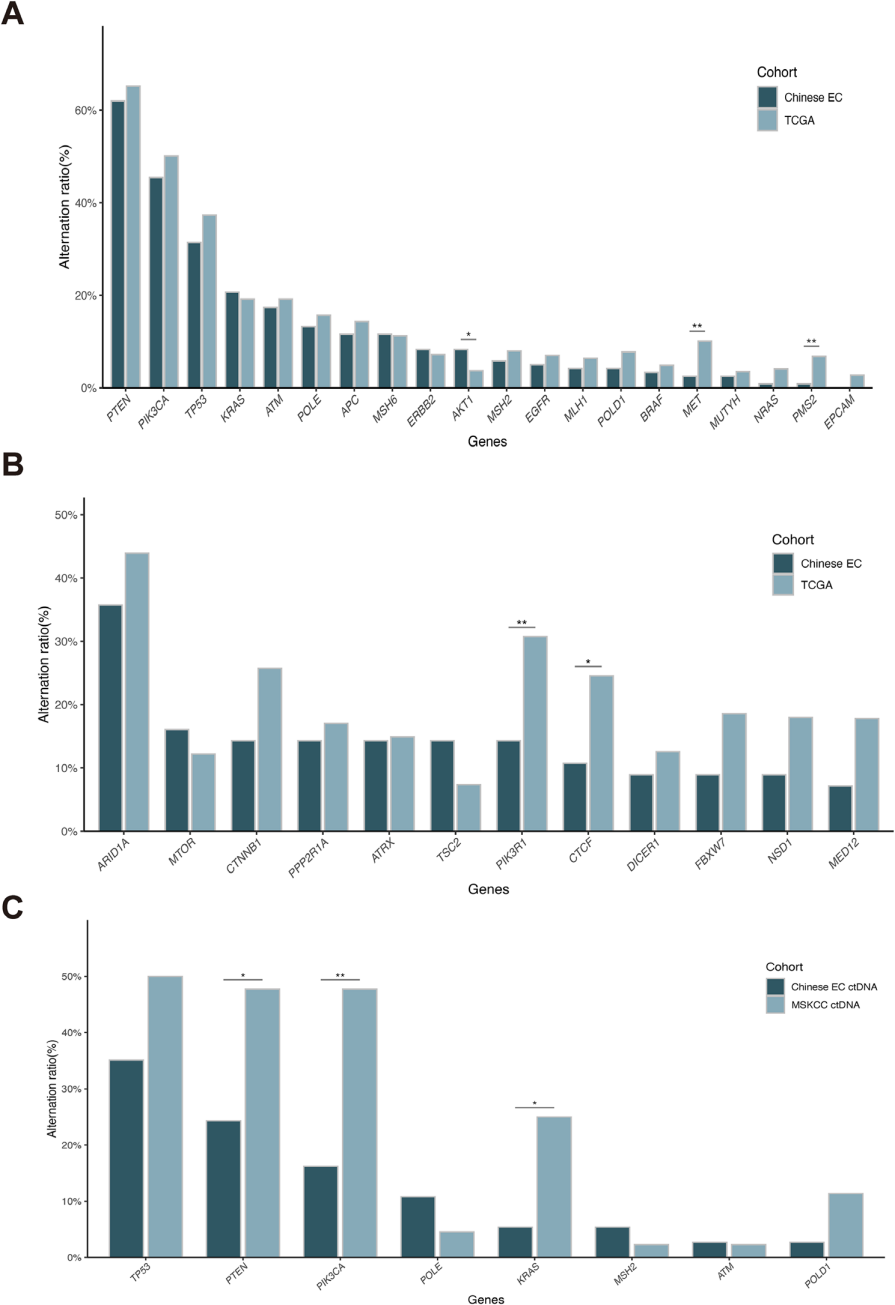
Differences in the prevalence of altered genes between Chinese and Western endometrial carcinoma cohorts. **A**, Comparison of the prevalence of 20 genes between tumor samples in Chinese cohort and the TCGA cohort. **B**, Comparison of the prevalence of top 20 genes tested in the expanded panel between tumor samples in Chinese cohort and the TCGA cohort. **C**, Comparison of the prevalence of genes between ctDNA samples in Chinese cohort and the MSKCC cohort. ctDNA: Circulating tumor DNA; * p < 0.05; **p < 0.01

Since we collected ctDNA samples in current study, it is important to consider that directly comparing the genomic feature regardless of sample type may lead to distorted outcomes. Therefore, we initially compared the prevalence of 20 EC genes between Chinese EC patients with tumor samples and TCGA-UCEC cohort. The result revealed a generally similar genomic profile among those 20 selected genes, with slight difference observed in *AKT1*, *MET* and *PMS2* (Fig. 3A). Additionally, when comparing the findings from tumor samples that underwent testing using the expanded panel testing to the TCGA-UCEC cohort, a higher altered frequency of *PIK3R1* and *CTCF* was identified in the TCGA cohort (Fig. 3B). We further compared the ge etic findings between our cohort and CPTAC cohort to assess the differences. The results revealed that all the aforementioned differences were diminished, only with the exception in *PIK3R1* (Supplementary Fig. 3). Furthermore, when comparing the genetic changes in ctDNA samples, we found a significantly higher prevalence of *PTEN*, *PIK3CA* and *KRAS* in MSKCC cohort (Fig. 3C). However, it is important to note that we observed similar genomic differences between local tumors and ctDNA samples (Supplementary Fig. 4). Considering the limited sample size of ctDNA in both our and the MSKCC cohort, we did not attribute these findings solely to the genomic difference in liquid samples between Chinese and Western cohorts.

### Clinically actionable alterations

**Fig. 4:**
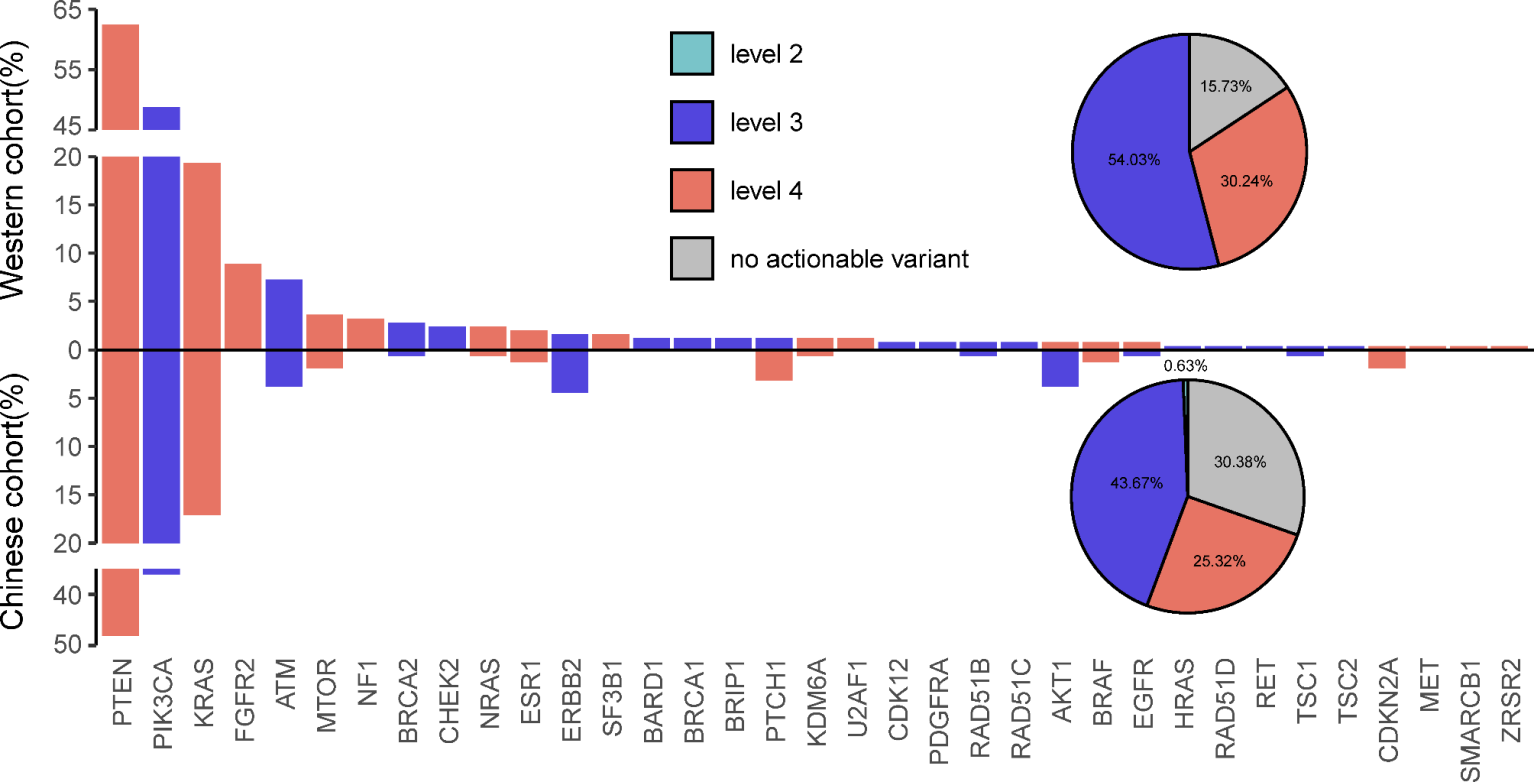
Comparison of actionable variants of endometrial carcinoma patients between the Chinese cohort and the Western cohort

In our cohort, we identified a total of 110 EC patients (69.6%) with actionable alterations (Fig. 4; Table 3). Among these patients, 51 individuals underwent testing with the expanded panel, while 59 patients were tested using the 20-gene panel, representing 60.7% (51/84) and 79.7% (59/74) of patients, respectively. We categorized all actionable alterations into four levels based on the OncoKB knowledge base. The vast majority of alterations fell into level 3 or 4, with only one exception being *ERBB2* amplification, which was classified as level 2. Furthermore, we observed that the prevalence of EC patients with actionable alterations was higher in the Western cohort compared to our cohort (84.3% vs. 69.6%, as shown in Fig. 4). This difference can be mainly attributed to the differences in *PTEN* and *PIK3CA* prevalence, which arise from the involvement of ctDNA samples in our cohort.

### Identifying the molecular subtypes of ***ARID1A*** wildtype EC patients

**Fig. 5:**
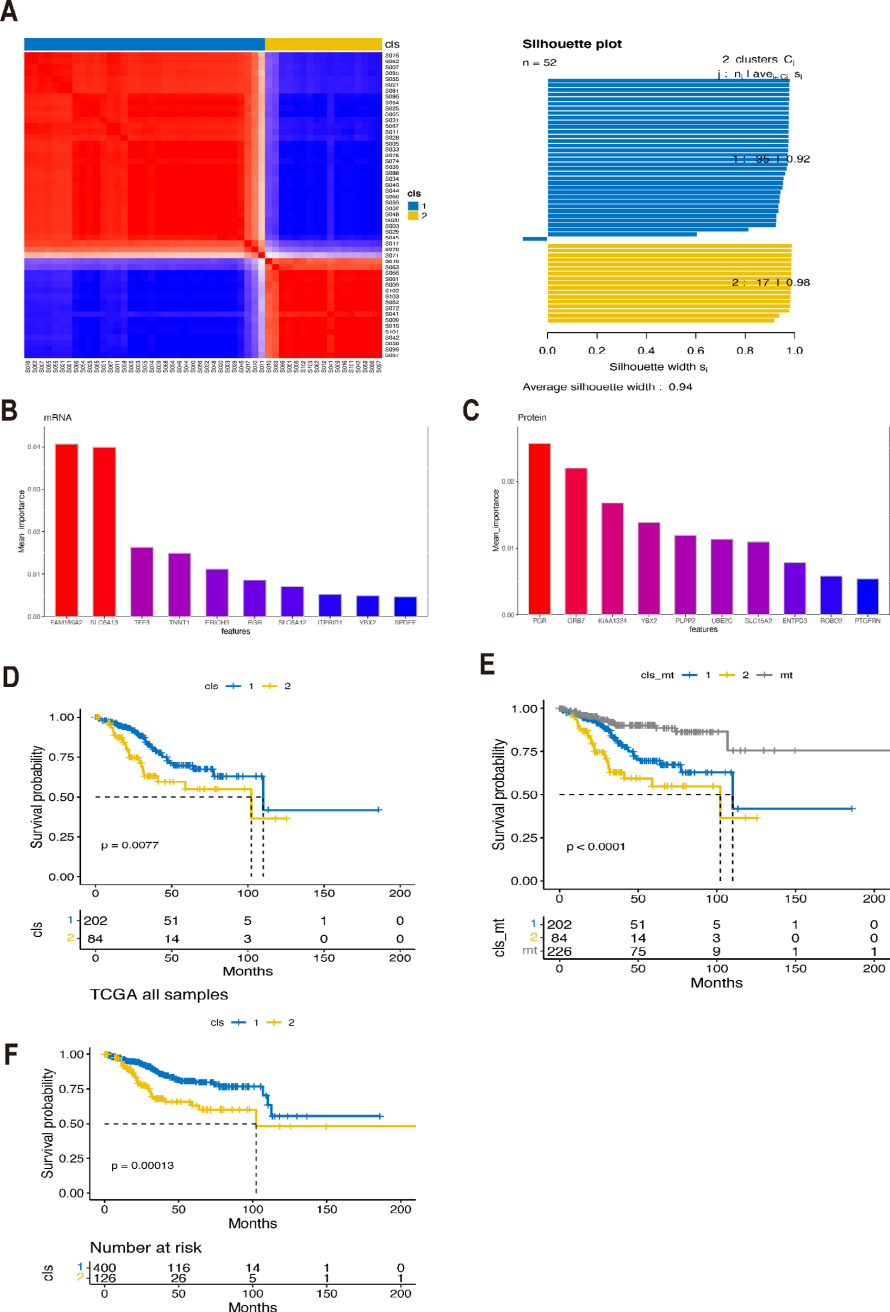
Identification the molecular subtypes of ARID1A-wt endometrial carcinoma patients. **A**, Consensus matrix of unsupervised clustering based on the integrative multi-omics data and identify the best cluster number with k = 2 in the CPTAC cohort. **B**, The silhouette width of unsupervised clustering based on SNF method in integrated omics data when k = 2. **C**, The most discriminative signatures of mRNA and protein selected by random forest algorithm. **D**. Kaplan-Meyer plot comparing patients within cluster 1 and cluster 2 from TCGA cohort. E. Kaplan-Meyer plot comparing patients within cluster 1, cluster 2 of ARID1A-wt subset and ARID1A-mt subset. F. Kaplan-Meyer plot comparing patients with high and low cluster-signature without considering their ARID1A status. Wt: wildtype; mt: mutated

Alteration in *ARID1A* is prevalent in both Chinese and western EC patients, and previous studies have associated it with a favorable prognosis in EC [20]. While it was initially recognized as a member of SWI/SNF chromatin remodeling complex, recent evidence also suggests its involvement in PI3K [21] and DDR [22] pathways. Given that, we utilized transcriptomic and proteomic data from the CPTAC cohort to investigate whether there existed molecular heterogeneity in *ARID1A*-wildtype EC patients. Through our analysis, we identified two distinct molecular clusters of ARID1A-wildtype EC based on these data (Fig. 5A). Subsequently, employing a random forest machine learning algorithm, we successfully identified ten mRNAs and ten proteins that exhibited the most prominent differences between these two clusters at the mRNA and protein levels, respectively (Fig. 5B&C). o validate the established molecular signatures associated with ARID1A-wildtype subtypes, we applied them to the TCGA cohort. In the TCGA cohort, we again identified two clusters within the ARID1A-wildtype EC group, and patients in cluster 2 displayed inferior overall survival compared to those in cluster (Fig. 5D). Furthermore, both of these clusters had worse prognoses than EC patients with *ARID1A* alterations (Fig. 5E). Interestingly, when we applied the same cluster signature derived from the CPTAC cohort to cluster EC patients in the TCGA cohort, irrespective of their ARID1A status, patients within cluster 2 also exhibited significantly shorter overall survival (Fig. 5F).

### Genomic and tumor microenvironment feature related to the molecular subtypes of ***ARID1A*** wildtype EC patients

**Fig. 6:**
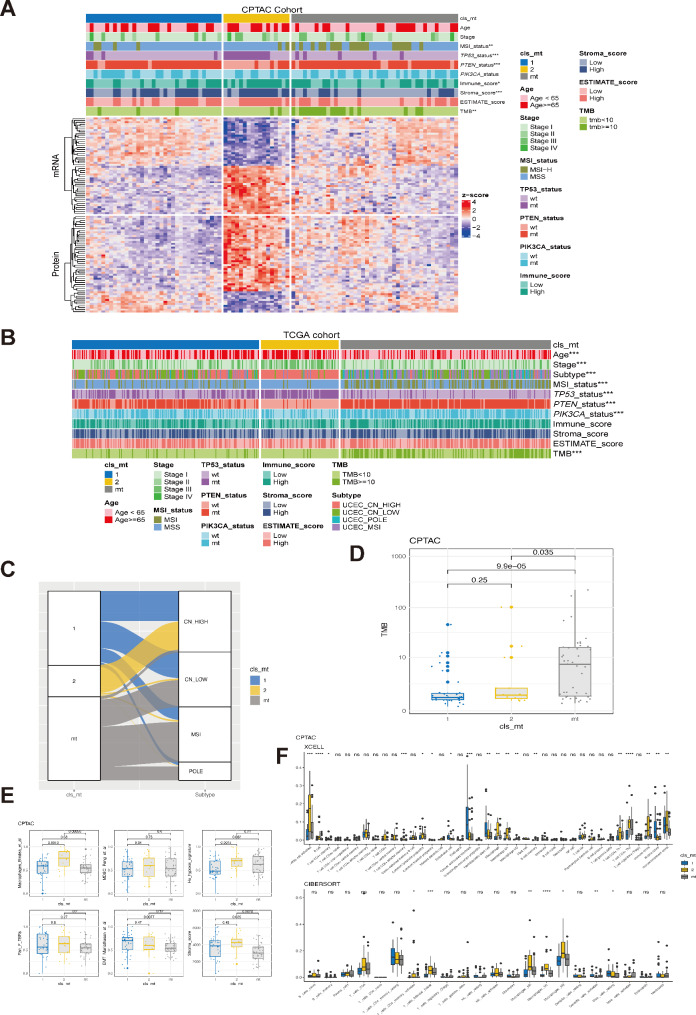
The molecular and tumor microenvironment feature related to ARID1A-wt clusters. **A**, Heatmap illustrated the clinical and molecular characteristics among cluster 1 and 2 and ARID1A-mt in endometrial carcinoma patients from CPTAC cohort. **B**, Heatmap illustrated the clinical and molecular characteristics among cluster 1 and 2 and ARID1A-mt in endometrial carcinoma patients from TCGA cohort. **C**, The distribution of TCGA molecular classification subtypes among cluster 1 and 2 and ARID1A-mt group. **D**, Difference in the tumor mutation burden (TMB) among cluster 1 and 2 and ARID1A-mt group. **E**, Distribution of tumor immunology related signatures among cluster 1 and 2 and ARID1A-mt group in CPTAC cohort, including Macrophages, MDSC, hypoxia signature, EMT signature, Pan F TBRs, and Stromal score. **F**, Difference in the abundance of tumor-infiltrated immune cells among cluster 1 and 2 and ARID1A-mt group in CPTAC cohort analyzed by XCELL (top panel) and CIBERSOFT (bottom panel)

To investigate the molecular and immunological characteristics of the two clusters, we conducted an analyisis of the genomic classification in both CPTAC and TCGA cohorts. Our findings demonstrated significant genomic differences between cluster 2 and cluster 1, as well as the ARID1A mutant groups. Specifically, cluster 2 exhibited a higher frequency of TP53 alterations but a lower prevalence of PTEN and PIK3CA alterations. Moreover, cluster 2 displayed higher immune, stromal, and ESTIMATE scores (Fig. 6A). These genomic differences were consistently overserved in the TCGA cohort (Fig. 6B). Moreover, we observed that cluster 2 was enriched with patients who were older and at more advanced stages. Notably, cluster 2 exclusively belonged to the CN-high subtype, which was consistent with the higher prevalence of *TP53* alteration in this group (Fig. 6C). However, there was no significant difference in the TMB level between the two clusters, both of which were significantly lower than that of patients with *ARID1A* alterations (Fig. 6D). Additionally, we found that cluster 2 was characterized by significantly higher levels of macrophages, hypoxia, and stromal score (Fig. 6E). The analysis of macrophages, regardless of their polarization status, and activated dendritic cells using XCELL and CIBERSORT also indicated higher levels in cluster 2 (Fig. 6F).

### Discussion

While Western EC patients have been extensively studied, there is still a lack of comprehensive characterization of the genomic traits in Chinese EC patients. To address this gap, we conducted genetic analysis on 158 samples obtained from Chinese EC patients using NGS technology. The obtained data was then compared with existing data from Western cohorts. The molecular classification of EC endometrial cancer has been found to be correlated with several genes, such as POLE, TP53, and MMR genes. Furthermore, the remaining genes included in the 20-gene panel have been identified as being associated with the treatment and prognosis of EC endometrial cancer [2, 4, 5, 23–25]. Thus, the 20 genes were selected for comprehensive analysis, and 85.4% of patients were proved to have at least one alteration in the 20 genes. By integrating the testing results from two panels, we observed that *PTEN*, *PIK3CA*, and *TP53* were the most commonly altered genes in Chinese EC patients. However, the prevalence of *PTEN* and *PIK3CA* alterations in our cohort was significantly lower compared to the TCGA-UCEC cohort. It is worth noting that a previous study involving 115 Chinese EC patients also reported PTEN (53.9%) and PIK3CA (46.1%) as the most frequently altered genes [26]. However, the prevalence of *TP53* was 7.8% that lower than our cohort (27.8%), which may be related to the difference in the pathological composition. Additionally, co-occurring in *PTEN* and *PIK3CA* was identified in our cohort, which is consistent with previous reports [26, 27].

Several frequently altered signaling pathways have been identified and proven to have roles in EC, as evidenced by studies such as TCGA and others [4, 5]. Alterations in the PI3K pathway are particularly prevalent in EC, occuring in 80–95% of EC cases [5, 28, 29]. In our cohort, which consisted of 84 samples tested using the expanded panel, we found that 69.0% of patients harbored PI3K pathway alterations, which is slightly lower than the findings reported in previous studies. The DDR pathway has gained increasing attention in cancer therapy due to the promising therapeutic effects of DDR pathway-targeting drugs. Therefore, genetic and genomic analysis of the DDR pathway has become a focal point of researc [30]. In our cohort, we identified DDR pathway alterations in 30.1% (26/84) of patients, with approximately half of these alterations occurring in the HR pathway. Furthermore, we observed that alterations in *MTOR*, *CTNNB1*, *ATRX*, *FAT1*, and *KMT2B* were more frequently observed in patients with DDR pathway alterations, suggesting an association between the mutation status of these genes and DDR pathway alterations.

Previous research has primarily focused on the association between germline variants and an increased risk of developing EC, particularly in relation to MMR and Lynch syndrome [24, 25]. However, the prevalence of pathogenic or likely pathogenic (P/LP) germline variants in Chinese EC patients remains unclear. In our study, we identified that 11% of Chinese EC patients carried P/LP germline variants, some of which were rarely reported before. Notably, we identified one Chinese EC patient with a deleterious germline variant in the STK11 gene, a known tumor suppressor gene associated with the AMPK and mTOR pathway. Pathogenic variants in this gene can lead to Peutz-Jeghers Syndrome, which increases the risk of developing hamartomatous polyps in various organs such as the digestive tract, breast, testicles, ovaries, lung, cervix, and uterus [31]. Another Chinese EC cohort also reported that 12.66% (10/79) of EC patients had deleterious germline variants, although these were not observed in 36 endometrial intraepithelial neoplasia patients [32]. The prevalence of deleterious germline variants in unselected Chinese EC patients is comparable to that in Western patients. In a study by Kari et al., 9.2% (35/381) of unselected Western EC patients were found to have P/LP variants, predominantly in genes involved in MMR (22/358) and HR pathways (8/358) [33]. Although EC is commonly associated with Lynch syndrome, our findings, along with previous studies, suggest the importance of conducting germline testing using expanded panels. Identifying germline variants is crucial not only for understanding the underlying causes of carcinogenesis and assessing the cancer risk for relatives but also for evaluating the patient’s treatment and prognosis. For example, germline BRCA1/2 variants are not only associated with increasing risk for serous/serous-like EC [34], but they also confer sensitivity to PARP inhibitors, which have been widely approved in other cancer types. Additionally, germline BRCA1/2 variants are also related to a distinct clinicopathologic entity that associated with unfavorable clinical outcomes [35].

The treatment of solid tumors based on matching actionable alterations to targeted therapies has resulted in significant improvements in outcomes of patients with advanced cancers, which has been comprehensively demonstrated by multiple umbrella and or basket trails [36]. In this study, 69.6% of patients with EC were identified with actionable alterations, although this proportion was lower compared to the Western cohort. Among these alterations, *ERBB2* amplification was identified as the only actionable alteration with level 2, suggesting potential benefit from Carboplatin-Paclitaxel-Trastuzumab therapy [37]. However, in our current study, we only identified one patient with significant ERBB2 amplification, which appears to be lower than the results reported in other datasets or previous studies [38]. Several factors may contribute to this discrepancy: (1) ERBB2 amplification is more commonly observed in serous EC, which was less prevalent in our cohort. (2) The standard methods for detecting ERBB2 (HER2) amplification, such as immunohistochemistry (IHC) and fluorescence in situ hybridization (FISH), may yield different results compared to NGS [39]. (3) Notably, 23.42% of patients in our study underwent genetic testing using ctDNA, which may have influenced the incidence of HER2 amplification [40]. (4) Furthermore, the difference in ethnic backgrounds between our cohort and Western cohorts may also contribute to this discrepancy. Apart from the actionable alterations enriched in the PI3K pathway, the majority of remaining alterations were observed in the DDR pathway. Although limited evidence supports the efficacy of PARP inhibitors in HR-deficient EC patients [41–44], the comprehensive approved indications and mechanism of PARP inhibitors justify their application in EC patients as well.

In 2013, TCGA introduced a classification system for endometrial cancer by identifying four molecular subtypes, which were found to be linked to the survival outcomes of EC patients [5]. Subsequently, other classification systems such as Proactive Molecular Risk Classifier for Endometrial Cancer (ProMisE) and TransPORTEC have been propose [23, 45, 46]. However, further research is still needed to determine whether more precise biomarkers or classification systems exist that can not only improve prognosis classification but also guide the selection of appropriate therapeutic regimens. *ARID1A*, a tumor suppressor gene involved in chromatin remodeling, is frequently detected in EC [47, 48]. Among the case tested using expanded panel, *ARID1A* alterations were detected in 27.4% of patients. Genomic alteration in *ARID1A* have been found to be correlated with its RNA and protein expression level in EC [49]. *ARID1A*-mutated EC exhibit decreased PgR transcription levels, which are associated with changes in the PgR enhancer region during early tumor development. This mutation has been implicated in the malignant transformation from atypical hyperplasia to EC [50, 51]. Considering that ARID1A alteration has been extensively associated with improved prognosis and enhanced efficacy in immunotherap [52], investigation the molecular heterogeneity in *ARID1A*-wildtype EC patients becames even more critical. The identified cluster 2, which was characterized by enrichment of CN-high/TP53-altered patients, exhibited the worst outcomes compared to cluster 1 and the ARID1A-mutated group. Notably, cluster 2 had significantly lower prevalence of PIK3CA and PTEN alterations, indicating a lack of changes in the PI3K signaling pathway. However, cluster 2 demonstrated distinct tumor immunology, including higher immune and stromal scores and increased presence of macrophages. Tumor-associated macrophages have been widely associated with poor prognosis, angiogenesis, and loss of PgR in EC [53, 54].

Our study had certain limitations. Firstly, to present a more comprehensive genomic landscape of Chinese EC, it would be necessary to improve this study by expanding the sample sizes. Simultaneously, approximately 46% of the patients included in this study underwent genetic testing using the 20-gene panel. While this panel encompasses the most common and crucial oncogenes/tumor suppressor genes associated with EC and allows for molecular classification, it is important to acknowledge that the limited gene spectrum may restrict our understanding of the complete genetic profile beyond these 20 genes. Despite ctDNA being widely considered as a surrogate for tumor tissue in genetic profiling, it is important to acknowledge the potential discordance between findings from these two sample types. In our current study, we did not perform genomic testing using ctDNA and tumor tissue samples from the same individuals. Due to the shortage of transcriptomic and survival data in our cohort, we were unable to validate the identified ARID1A-wt subtypes. Thus, further validation in a local cohort using prospective clinical samples and data is warranted.

## Conclusion

In summary, our study identified the genomic profile of Chinese patients with EC, providing valuable insights for potential therapy selection in EC patients. Additionally, we discovered the molecular heterogeneity in ARID1A-wildtype EC patients, which revealed unique genomic and immunological features. These findings contribute to a better understanding of EC and have implications for personalized treatment approaches.

**Table 1:** Clinical characteristics of 158 Chinese patients with endometrial carcinoma

Characteristics		NO. (%)	NO. of patients took the 20-genes panel testing	NO. of patients took the expanded panel testing
Total patients		158 (100%)	74 (46.84%)	84 (53.16%)
Median age (range)		56 (25–80)	56 (25–78)	54 (32–80)
Histologic subtypes				
	endometrioid	134 (84.81%)	70 (44.30%)	64 (40.51%)
	carcinosarcoma	15 (9.49%)	2 (1.27%)	13 (8.23%)
	serous	5 (3.16%)	1 (0.63%)	4 (2.53%)
	clear cell	2 (1.27%)	1 (0.63%)	1 (0.63%)
	undifferentiated carcinomas	1 (0.63%)	0 (0%)	1 (0.63%)
	Endometrioid & clear cell	1 (0.63%)	0 (0%)	1 (0.63%)
Sample type				
	Tissue	121 (76.58%)	65 (41.14%)	56 (35.44%)
	Blood	37 (23.42%)	9 (5.70%)	28 (17.72%)
Stage				
	I	6 (3.80%)	4 (2.53%)	2 (1.27%)
	II	109 (68.99%)	57 (36.08%)	52 (32.91%)
	III	11 (6.96%)	3 (1.90%)	8 (5.06%)
	IV	32 (20.25%)	10 (6.33%)	22 (13.92%)

**Table 2:** Pathogenic or likely pathogenic germline variants in Chinese EC patients

Patients No.	Gene	Mutation Type	Nucleotide Change	AA Change
#1	*BRCA2*	nonsynonymous	c.8168 A > G	p.Asp2723Gly
#2	*BARD1*	frameshift-insertion	c.1350_1351insT	p.Gly451fs
#3	*MLH1*	splice	c.677 + 1G > A	c.677 + 1G > A
#4	*MSH2*	stopgain	c.1216 C > T	p.Arg406Ter
	*RAD50*	frameshift-deletion	c.80_83delTCTT	p.Phe27fs
#5	*MSH6*	frameshift-insertion	c.3261dupC	p.Phe1088fs
#6	*MLH1*	nonsynonymous	c.350 C > T	p.Thr117Met
#7	*MLH1*	frameshift-deletion	c.274_283delGCCAGTATTT	p.Ala92fs
#8	*STK11*	frameshift-insertion	c.842dupC	p.Leu282fs
#9	*MLH1*	frameshift-deletion	c.526delA	p.Ile176fs
#10	*BRCA2*	frameshift-deletion	c.5164_5165delAG	p.Ser1722fs

**Table 3:** Actionable alterations identified in Chinese EC cohort

OncoKB level of evidence	Altered genes	Mutational type		No of patients		Percentage (%)		NO. of patients took 20-genes panel testing	Percentage (%)	NO. of patients took expanded panel testing	Percentage (%)
Total			110		69.6		59		79.7	51	60.7
2	*ERBB2*	amplification	1		0.6		0		0.0	1	1.2
3	*AKT1*	Oncogenic	6		3.8		2		2.7	4	4.8
3	*ATM*	Oncogenic	6		3.8		5		6.8	1	1.2
3	*BRCA2*	Oncogenic	1		1.2		NA		NA	1	1.2
3	*EGFR*	Oncogenic	1		0.6		1		1.4	0	0.08
3	*ERBB2*	Oncogenic	6		3.8		3		4.1	3	3.8
3	*FLT3*	Oncogenic	1		1.2		NA		NA	1	1.2
3	*IDH1*	Oncogenic	1		1.2		NA		NA	1	1.2
3	*PIK3CA*	amplification	1		0.6		0		0.00	1	1.2
3	*PIK3CA*	Oncogenic	56		35.4		31		41.9	25	29.8
3	*RAD51B*	Oncogenic	1		1.2		NA		NA	1	1.2
3	*TSC1*	Oncogenic	1		1.2		NA		NA	1	1.2
4	*AKT1*	Oncogenic	1		1.2		1		1.4	0	0.0
4	*BRAF*	Oncogenic	2		1.3		1		1.4	1	1.2
4	*CDKN2A*	Oncogenic	3		3.6		NA		NA	3	3.6
4	*EGFR*	Oncogenic	1		0.6		1		1.4	0	0.0
4	*ESR1*	Oncogenic	2		2.4		NA		NA	2	2.4
4	*KDM6A*	Oncogenic	1		1.2		NA		NA	1	1.2
4	*KRAS*	Oncogenic	27		17.1		19		25.7	8	9.5
4	*MTOR*	Oncogenic	3		3.6		NA		NA	3	3.6
4	*NRAS*	Oncogenic	1		0.6		1		1.4	0	0.0
4	*PTCH1*	Oncogenic	5		5.6		NA		NA	5	6.0
4	*PTEN*	Oncogenic	76		48.1		45		60.8	31	36.9

### Electronic supplementary material

Below is the link to the electronic supplementary material.

Supplementary Material 1

Supplementary Material 2

## Data Availability

The datasets generated and/or analysed during the current study are available in the National Genomics Data Center, China National Center for Bioinformation/Beijing Institute of Genomics, Chinese Academy of Sciences under the access number is PRJCA016701 (https://ngdc.cncb.ac.cn/bioproject/browse/PRJCA016701).
