# Supplementary material for: Genomic profile of Chinese patients with endometrial carcinoma
Source: BMC Cancer. 2023 Sep 20;23:888. doi: 10.1186/s12885-023-11382-4 (PMC10512642; doi:10.1186/s12885-023-11382-4)

Supplementary figure legend

Supplementary figure 1 The lollipop plot shows the genomic distribution of *PTEN* (A), *POLE* (B), *TP53* (C), and *PIK3CA* (D). The grey bar represents the entire protein with the different amino acid positions. The length of the grey lines indicates the number of alterations detected at the specified position, and the colored circles on the grey bar represent the corresponding alteration types. The colored boxes are different functional domains.

Supplementary figure 2 Comparisons of the prevalence of mutated genes between EC patients with and without alteration in DDR pathway.

Supplementary figure 3 Genomic difference between our and CPTAC endometrial carcinoma cohort. A, Comparison of the prevalence of 20 genes between our and CPTAC endometrial carcinoma cohort. B, Comparison of the prevalence of top 20 genes tested in the expanded panel between our and CPTAC endometrial carcinoma cohort. * $p<0.05$; ** $p<0.01$, *** $p<0.001$.

Supplementary figure 4 Genomic difference between tumor and ctDNA samples from local endometrial carcinoma cohort. ctDNA: Circulating tumor DNA; * $p<0.05$; ** $p<0.01$, *** $p<0.001$.

Supplementary figure 1

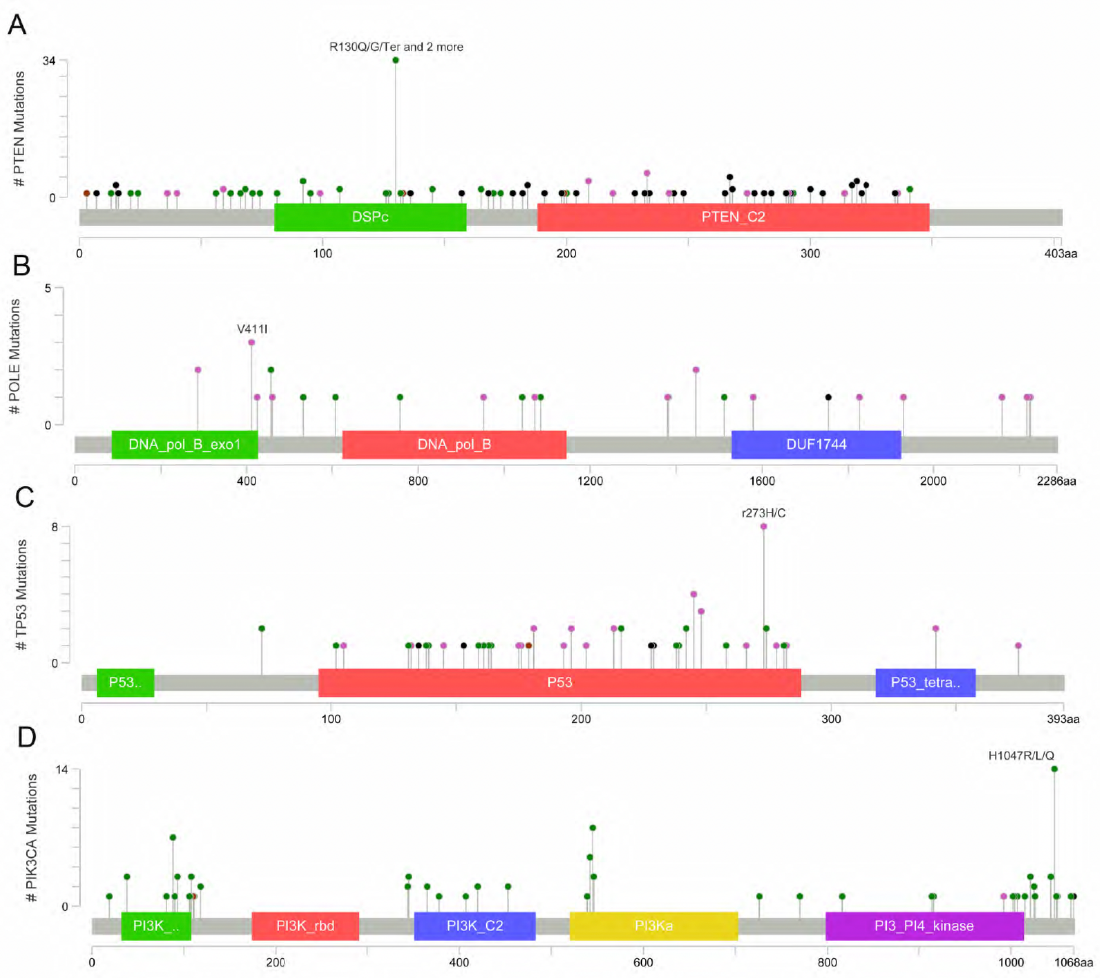

Supplementary figure 2

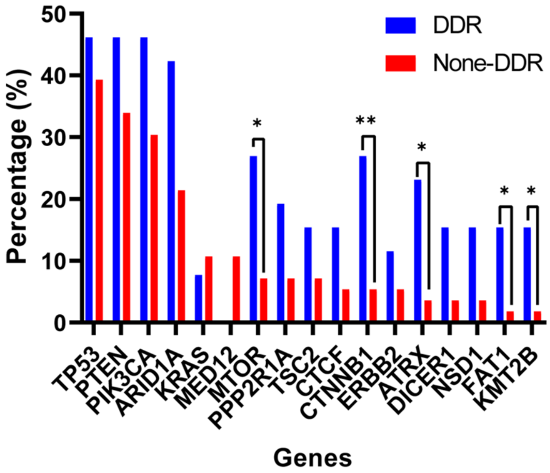

Supplementary figure 3

A

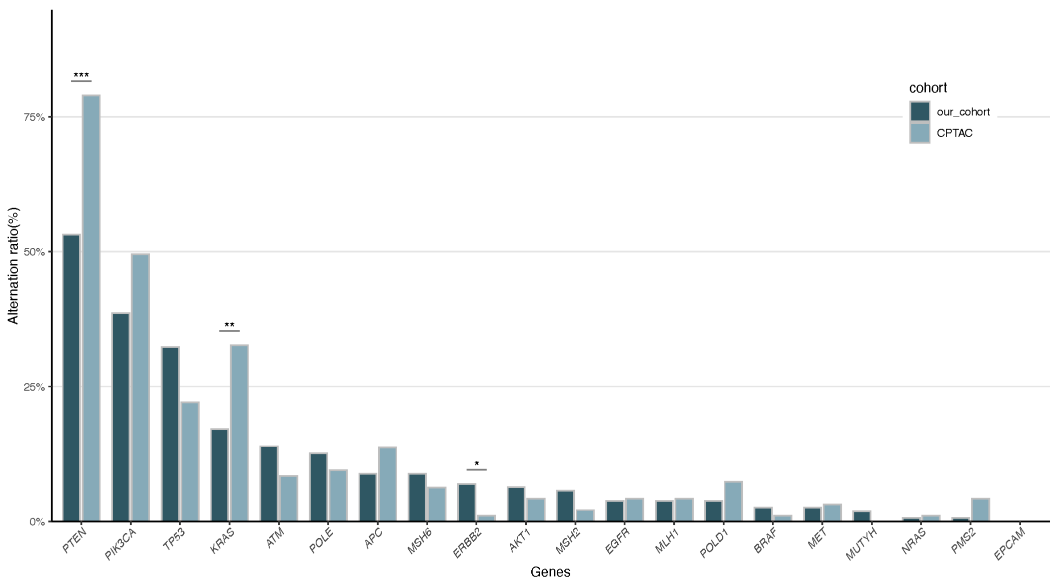

B

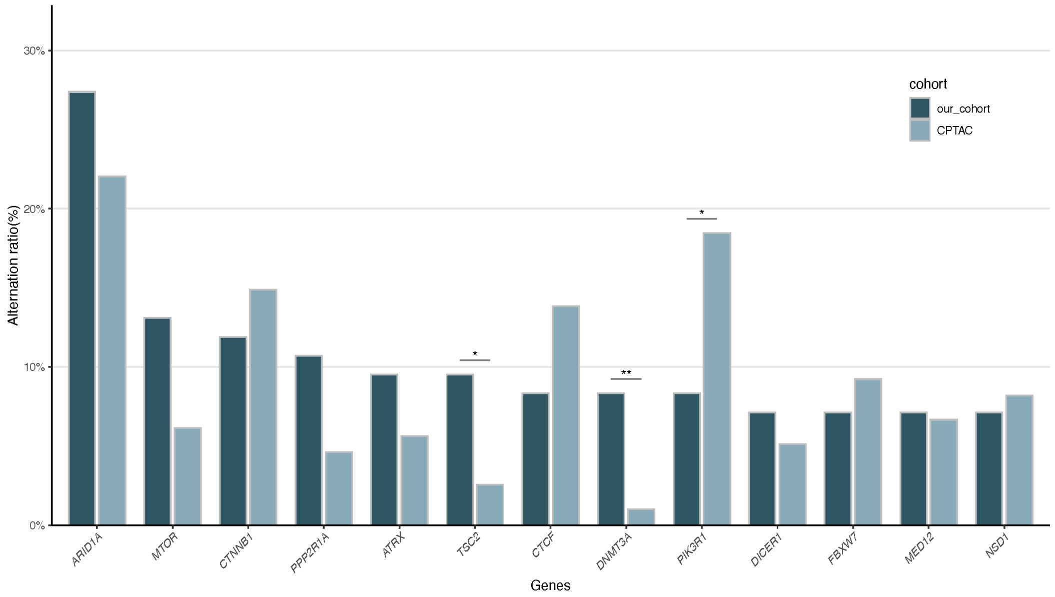

Supplementary figure 4

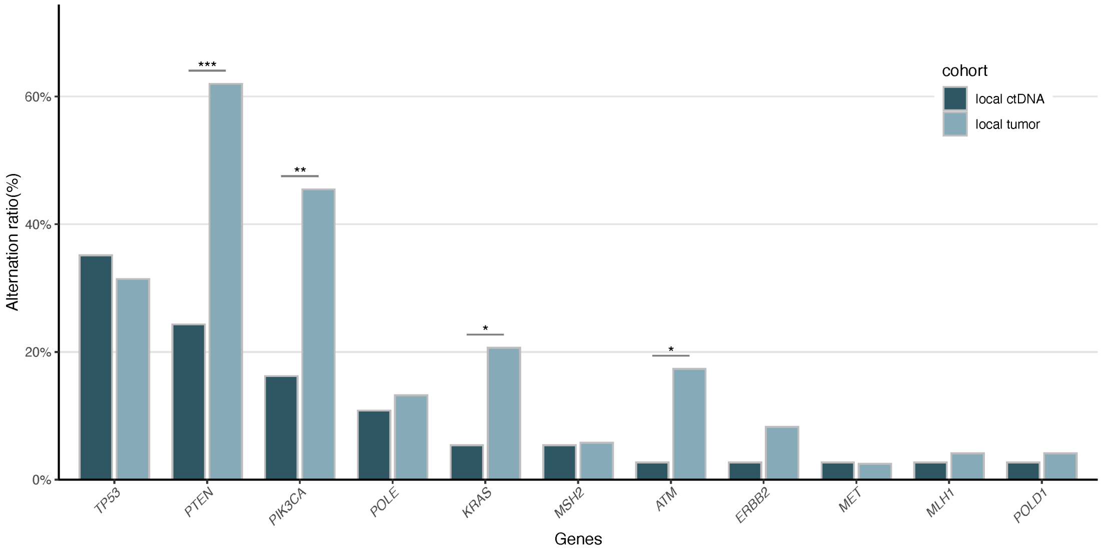

Supplement: Supplementary file 1 — Supplementary Material 1 [file 12885_2023_11382_MOESM1_ESM.pdf]
